# Inadvertencies

**Published:** 1859-08

**Authors:** 


### INADVERTENCIES.

To stand in the front door of a city car, while all dusty, perspiring and begrimed, with the fumes of the filthy carcass blowing full in the faces of the thirty passengers in the rear.

To open the window next you in a rail car, without consulting the convenience of the passenger behind you.

To stop for conversation in the aisle or doorway of a church or other building, thus preventing the passage of dozens of others.

